# Treatment of Dyspeptic Headache

**Published:** 1848-05

**Authors:** George Chaplin Child


### SELECTIONS

FROM

### AMERICAN AND FOREIGN JOURNALS.

---

*Treatment of Dyspeptic Headache.* By GEORGE CHAPLIN CHILD, M. D.—[We have extracted the following practical remarks from a work which we shall further analyze in our Report. They embody the views of a man who has evidently had considerable opportunities of studying dyspepsia, and its various forms. He observes that:]

There is, perhaps, no single dyspeptic symptom that causes greater suffering than headache, or of which patients are more anxious to be relieved. Many apply on account of headache solely, without suspecting that it depends on a disordered stomach; and others, although aware of this, know also that the radical cure of their complaint is likely to occupy a considerable time, and they would, therefore, feel grateful if some palliative could be devised meanwhile to lessen present suffering.

The headache following a debauch in eating and drinking, seldom requires any special treatment, provided the stomach were previously in a sound state. The erythema of the mucous membrane subsides within forty-eight hours, and with it the symptomatic headache. In slight cases, if the headache be attended with collapse and debility, a little food or a dose of sal volatile in some aromatic infusion often does good.

After gross dietetic errors in persons whose stomach is already in a morbid state, the headache is apt to last for several days, and to be accompanied with various febrile symptoms. The gastric mucous membrane is, in fact, acutely inflamed; and should there be nothing to contra-indicate it, the best treatment is to apply six or ten leeches to the epigastrium. When the headache has persisted for any considerable period, it may bring on determination of blood, and it may then be advisable to cup or leech the nape of the neck, or to apply leeches to the feet, and afterward promote bleeding and revulsion by hot pediluvia.

In violent gastric and bilious headache, and in those where there is throbbing, cold applications, as bags of ice, are extremely useful, and at the same time, grateful to the patient; or the forehead may be occasionally moistened with a lotion containing one part of acetic ether diluted with four parts of water; ether alone, to produce the full refrigerant effect, or combined with some anodyne, as tincture of aconite.

As the primary irritation in the stomach arises from food acting on morbidly-sensitive nerves, it is always necessary to remove the exciting cause, as far as practicable, by enjoining a spare and bland diet. The undue sensibility of the stomach, as well as the predisposition to secondary pain in the nerves of the head, ought likewise to be temporarily blunted by anodynes. Of these, the extract of belladonna (gr. $\frac{1}{6}$ to $\frac{1}{4}$), or of henbane (gr. iij.), with ipecacuanha (gr. $\frac{1}{2}$), made into a pill with confection of roses, and given thrice daily, is the most useful.

In London, and probably in all large towns, the most frequent cause of headache is bilious congestion, to relieve which no remedy equals an emetic, as it strikes at the root of the disorder by thoroughly unloading the liver. This plan of treatment is nearly always admissible, for it is a mistake to suppose that an occasional emetic permanently weakens the stomach even of a dyspeptic. An emetic, however, is now an unfashionable remedy, and, what may be fairly allowed to have more weight, it is certainly a most disagreeable one. When, therefore, from this or any other reason, it cannot be given, our next best plan is to excite brisk action of the bowels, and thus drain off from the liver the suppressed or pent-up bile. At the same time, patients should be warned that drastic purgatives attain the proposed end only by the irritation they produce, first in the stomach and then in the duodenum and bowels, and that this excitement cannot fail to do mischief in cases of dyspepsia. Not only do drastic purgatives cause more irritation than an emetic, but they also unload the liver less effectually. The action of the latter is soon over, and one of its chief advantages is, that it obviates the necessity of giving large quantities of strong physic. All purgative medicines do not seem equally efficacious in procuring the downward evacuation of the bile. The best are calomel (gr. iv. to vj.), compound extract of colocynth (gr. x.), compound powder of jalap (ʒss.), and sulphate of manganese (gr. x.), in half a tumblerful of water. When this last medicine does not produce sickness, it may be given for several days in succession without inconvenience, as its cholagogue power is great in comparison to its drastic properties.

The headache, observed in some dyspeptics, may be either entirely excited, or aggravated, by uterine irritation. I have the notes of several cases of indigestion before me, wherein the patients were not liable to headache at all, except just before the monthly periods. The same fact may be remarked in mennorrhagia, and in other diseases of the uterus attended with irritation. If amenorrhea be conjoined with dispepsia, the headache will probably be worst at the time when the catamenia ought to appear, which is often marked by what is called a molimen, or effort of nature to bring on the secretion, denoted by feverishness and an increase in the uneasy sensation usually felt—namely, pain in the back, abdomen, etc. The application of from two to six leeches to the groin, at this particular period, often causes the discharge actually to appear, and in other cases proves so good a substitute for it, as to relieve nature, and cut short the headache. Warm stupes across the abdomen ought to be perseveringly employed.

When headache is complicated with plethora, or determination of blood to the head, leeching or cupping at the nape of the neck may be necessary. The excess of blood, however, is often local only; or, on the contrary, the absolute quantity of it in the body may even be below the healthy standard.—In such cases, it may be of consequence to avoid depletion, and it will generally be found that the lost balance may be corrected in the circulation by dry cupping and blisters, or other modes of counter-irritation applied to the epigastrium, together with synapisms to the legs, and hot pediluvia.—*Ranking's Abstract.*

